# Starch-Coated Superparamagnetic Fe_3_O_4_ Nanoparticles: From Physicochemical Characterization to Cytogenetic Assessment in *Triticum aestivum* L.

**DOI:** 10.3390/nano16140886

**Published:** 2026-07-18

**Authors:** Mihaela Racuciu, Lucian Barbu-Tudoran, Marian Grigoras, Florin Brinza, Simona Oancea, Dorina Creanga

**Affiliations:** 1 Environmental Sciences and Physics Department, Faculty of Sciences, Lucian Blaga University of Sibiu, Dr. I. Ratiu Str., no. 5–7, 550012 Sibiu, Romania; 2 Electron Microscopy Integrated Laboratory, National Institute for R&D of Isotopic and Molecular Technologies, Donat Str., no. 67–103, 400293 Cluj-Napoca, Romania; lucian.barbu@itim-cj.ro; 3 Electron Microscopy Laboratory “Prof. C. Craciun”, Faculty of Biology and Geology, Babes-Bolyai University, Clinicilor Str., no. 5–7, 400006 Cluj-Napoca, Romania; 4 National Institute of Research and Development for Technical Physics, 47, Mangeron Blvd., 700050 Iasi, Romania; mgrigoras@phys-iasi.ro; 5 Faculty of Physics, “Alexandru Ioan Cuza” University, Carol I Blvd., 11, 700506 Iasi, Romania; fbrinza@uaic.ro (F.B.); mdor@uaic.ro (D.C.); 6 Agricultural Sciences and Food Engineering Department, Lucian Blaga University of Sibiu, Dr. I. Ratiu Str., no. 7–9, 550012 Sibiu, Romania; simona.oancea@ulbsibiu.ro

**Keywords:** iron oxide nanoparticles, starch coating, superparamagnetic, mitotic index, chromosomal aberrations

## Abstract

Iron oxide-based nanomaterials have attracted considerable interest owing to their unique magnetic properties and potential biomedical and environmental applications. In this study, starch-coated superparamagnetic Fe_3_O_4_ nanoparticles (Sta-MNP) were synthesized and comprehensively characterized using electron microscopy (TEM, SEM), energy-dispersive X-ray spectroscopy (EDS), X-ray diffraction (XRD), vibrating sample magnetometry (VSM), attenuated total reflectance Fourier-transform infrared spectroscopy (ATR-FTIR), and nanoparticle tracking analysis (NTA). The results confirmed the formation of a magnetite-based iron oxide nanoparticles sample with a median physical diameter of 12.24 nm, superparamagnetic behavior with a saturation magnetization of 59.81 emu/g, and effective starch coating on the nanoparticle surface. The biological effects of Sta-MNP were assessed in *Triticum aestivum* L. using the mitotic index (MI) and aberration index (AI) as cytogenetic endpoints, respectively. Exposure-induced concentration-dependent increases in both parameters across the tested volume fractions (0–200 µL/L), suggesting a significant interaction between Sta-MNP and dividing cells. Overall, this study provides a comprehensive physicochemical profile of starch-coated magnetite nanoparticles and demonstrates their potential cytogenetic impact in a plant model system, supporting further investigation of their environmental interactions and potential agricultural applications.

## 1. Introduction

The rapid development of nanotechnology has enabled the design of advanced materials with integrated and tunable functionalities. Multifunctional nanocomposites have emerged as promising materials for biological applications [1,2]. These systems combine two or more distinct components, typically an inorganic nanoscale phase and an organic shell or polymeric matrix, resulting in materials that exhibit synergistic physicochemical and biological properties beyond those of their individual constituents [3]. Such multifunctionality is especially relevant in environmental applications, where materials must simultaneously ensure stability and controlled interactions with plant systems, including uptake, translocation, and bioavailability [4].

Magnetic nanoparticles have been widely investigated owing to their unique combination of properties, including superparamagnetism, redox activity, and relatively low toxicity compared to other metal-based nanostructures [5,6]. These characteristics make them suitable for a broad range of applications, from biomedical systems to environmental and agricultural technologies [7]. In particular, the presence of Fe^2+^/Fe^3+^ redox couples enable participation in oxidative processes that can influence biological systems at the cellular level, including the modulation of enzymatic activity and induction of oxidative stress [8].

However, bare magnetite nanoparticles tend to aggregate owing to magnetic dipole–dipole interactions and high surface energy, which can significantly alter their physicochemical behavior and limit their applicability in biological environments [9]. To overcome these limitations, surface functionalization and stabilization using biocompatible polymers have been extensively explored [10]. Among these, starch has gained increasing attention as a natural, biodegradable, and non-toxic polysaccharide that can act as a stabilizing agent and functional component in nanocomposite systems [11]. Through its hydroxyl-rich structure, starch can interact with nanoparticle surfaces, improving colloidal stability, modulating dispersion, and influencing interactions between nanoparticles and biological systems [12].

The integration of iron oxide nanoparticles within a starch matrix can lead to the formation of multifunctional nanocomposites that combine magnetic responsiveness, redox activity, and polymer-mediated control over nanoparticle behavior in complex environments [1,5]. In addition to enhancing stability, polymeric components may play a critical role in regulating nanoparticle bioavailability, transport, and interactions with cellular structures, thereby directly impacting biological responses [13]. Compared to synthetic stabilizers, starch offers enhanced biodegradability and environmental compatibility, which are particularly advantageous in agricultural applications [14]. Moreover, its polysaccharidic structure may facilitate interactions with plant cell walls and influence the uptake, transport, and bioavailability of nanoparticles.

Although starch-coated Fe_3_O_4_ nanoparticles have attracted increasing interest due to their improved colloidal stability, biocompatibility, and environmental compatibility, previous studies have primarily explored their applications in photocatalysis and biomolecule separation [11,12]. In plant systems, iron oxide nanoparticles have been increasingly investigated for agricultural applications, including growth stimulation, nutrient delivery, and stress modulation [15]. However, most studies have focused on physiological and biochemical responses, whereas the cytogenetic effects of starch-stabilized Fe_3_O_4_ nanoparticles remain largely unexplored. Owing to their redox-active surfaces, magnetite nanoparticles may promote the generation of reactive oxygen species (ROS) through Fenton-like reactions, potentially leading to oxidative damage at the cellular and subcellular levels [16]. These processes may compromise DNA integrity, interfere with mitotic spindle function, and ultimately induce chromosomal aberrations and altered mitotic progression. Cytogenetic analysis, including the evaluation of the mitotic index, mitotic phase distribution, and chromosomal aberrations, provides valuable information on mitotic progression and chromosomal integrity, which are important indicators for assessing the cytogenetic safety of nanomaterials in plant systems [17].

Plant cytogenetic bioassays are widely used in environmental monitoring and toxicity assessment because they provide a sensitive, simple, and cost-effective approach for detecting cytotoxic and genotoxic effects induced by environmental contaminants. Among these, the Triticum aestivum root meristem assay is recognized as a useful model for evaluating mitotic activity, chromosomal aberrations, and DNA damage, and has been successfully applied in toxicity studies involving various classes of pollutants, including engineered nanomaterials [18,19]. These assays provide valuable information for the preliminary identification of genotoxic hazards and complement the overall safety assessment of emerging contaminants [20].

Therefore, there is a need for studies integrating comprehensive physicochemical characterization of starch-stabilized Fe_3_O_4_ nanoparticles with cytogenetic evaluation in plant systems, allowing relationships between nanoparticle physicochemical properties and cytogenetic responses in plant cells to be explored.

In this study, starch-stabilized magnetite nanoparticles (Sta-MNP) were synthesized and investigated as multifunctional nanocomposite systems. Comprehensive physicochemical characterization was performed using transmission electron microscopy (TEM), X-ray diffraction (XRD), vibrating sample magnetometry (VSM), Fourier-transform infrared spectroscopy (FTIR), and nanoparticle tracking analysis (NTA). The biological impact of the obtained nanocomposites was evaluated using cytogenetic assays in wheat (*Triticum aestivum* L.), with particular emphasis on mitotic activity and chromosomal stability.

Understanding these relationships may provide valuable insights into the factors governing nanoparticle–plant interactions and their cytogenetic consequences. Accordingly, the results are discussed in relation to the physicochemical properties of the nanoparticles and their biological effects, with particular emphasis on identifying possible relationships between nanoparticle characteristics and cytogenetic responses in plant cells.

## 2. Materials and Methods

### 2.1. Chemical Reagents

Ferric chloride hexahydrate (FeCl_3_ × 6H_2_O), ferrous chloride tetrahydrate (FeCl_2_ × 4H_2_O), 25% ammonium hydroxide (NH_4_OH), and soluble starch were sourced from Merck (Darmstadt, Germany) as analytical-grade reagents and used without any additional purification. Hydrochloric acid (HCl, 37%) was obtained from J.T. Baker Chemical Company (Deventer, The Netherlands). Absolute ethanol and glacial acetic acid were purchased from Chim Reactiv SRL (Bucharest, Romania).

### 2.2. Synthesis of Sta-MNP Aqueous Suspension

Starch-stabilized magnetic nanoparticles (Sta-MNPs) were synthesized via a modified co-precipitation method based on the controlled reaction of Fe^2+^ and Fe^3+^ ions in an alkaline medium, using ammonia as a precipitating agent, following a protocol adapted from [21,22]. The synthesis was carried out at 60 °C, followed by stabilization in a colloidal system using soluble starch, (C_6_H_10_O_5_)_n_, primarily via electrostatic interactions. Two aqueous precursor solutions were prepared at 60 °C. The first solution contained 0.021 mol FeCl_2_ × 4H_2_O (≈0.055 M) dissolved in 380 mL of distilled water, and the second solution contained 0.0386 mol FeCl_3_ × 6H_2_O (≈0.10 M) dissolved in 380 mL of distilled water. The solutions were magnetically stirred at a constant temperature (60 °C), and subsequently, 40 mL of 25% NH_4_OH was added dropwise at a controlled rate of 0.1 mL/s to induce precipitation of the iron oxide phase.

The resulting ferrophase was magnetically separated using a permanent magnet and allowed to settle at the bottom of the reaction vessel. The supernatant was removed, and the precipitate was washed with approximately 1 L of distilled water at ~30 °C to eliminate residual by-products such as iron hydroxides and unreacted precursor species.

The final product, appearing as a black wet ferrophase (~50 mL), was subsequently mixed with a starch solution obtained by dissolving 0.7 g of starch in 30 mL of distilled water at 60 °C. The mixture was mechanically stirred at 800 rpm for 75 min to ensure efficient stabilization. The concentration of the resulting aqueous suspension of starch-stabilized magnetic nanoparticles was estimated to be approximately 30 mg/L.

### 2.3. Characterization Methods of Sta-MNPs

Transmission electron microscopy (TEM) was employed to assess the nanoparticle morphology and size using samples prepared by depositing diluted suspensions onto carbon-coated copper grids (400 mesh), followed by drying under ambient conditions. Imaging was performed using a Hitachi HD 2700 CFEG STEM device (Hitachi, Tokyo, Japan) operated at an accelerating voltage of 200 kV. The elemental compositions of the Sta-MNP samples were determined using energy-dispersive X-ray spectroscopy (EDS).

Structural characterization was performed by X-ray diffraction (XRD) using a Shimadzu 600 XRD (Shimadzu, Kyoto, Japan) with Cu–Kα radiation (λ = 0.15418 nm) in Bragg–Brentano geometry within the 2θ range of 20–80°. The average crystallite size (D_hkl_) was derived from the diffraction data using the Debye–Scherrer equation—the relation (1) [23] is:

(1) $$D_{hkl}=\frac{0.9\cdot \lambda }{\beta \cdot cos\theta },$$

where λ denotes the X-ray wavelength, *θ* the Bragg angle, and β the full width at half maximum (FWHM) of the selected peak. The lattice parameter (a) was calculated according to relation (2) [24], considering the Miller indices (hkl), which typically correspond to the most intense (311) peak, and the associated interplanar distance d_hkl_.

(2) $$\frac{1}{d_{hkl}^{2}}=\frac{h^{2}+k^{2}+l^{2}}{a^{2}},$$

The interplanar distance was calculated by means of relation (3):

(3) $$d_{hkl}=\frac{\lambda }{2\cdot sin\theta_{hkl}}\;.$$

Magnetic measurements were conducted at room temperature (22 °C) via vibrating sample magnetometry (VSM) using a MicroMag Model 2900/3900 magnetometer (Lake Shore Cryotronics, Inc., Westerville, OH, USA). This technique provided the magnetization curves, saturation magnetization values, and coercive field of Sta-MNP. Based on these data, and assuming a spherical nanoparticle geometry, the mean magnetic core diameter (d_M_) was estimated using the Langevin model by means of relation (4) [25]:

(4) $$d_{M}^{3}=\frac{18\cdot K_{B}\cdot T}{\pi \cdot \mu_{0}\cdot M_{S}\cdot m_{S}}{\left(\frac{dM}{dH}\right)}_{H\to 0},$$

where K_B_ represents Boltzmann’s constant, M_S_ is the measured saturation magnetization of the sample, and m_S_ corresponds to the saturation magnetization of bulk magnetite (0.48 × 10^6^ A/m) [26]; T is the environmental temperature and μ_0_ is the magnetic permeability of vacuum.

The hydrodynamic size distribution and particle concentration in the suspension were determined using nanoparticle tracking analysis (NTA). Measurements were performed using a NanoSight LM20 device (NanoSight Ltd., Wiltshire, UK) equipped with a CCD camera operating at 30 frames per second and a red laser source. The Sta-MNP samples were analyzed at 22 °C at a dilution factor of 10^4^. Particle size distributions were obtained using NTA software (version 3.2) employing finite track length analysis (FTLA) for improved accuracy.

Vibrational properties were analyzed using attenuated total reflectance Fourier-transform infrared spectroscopy (ATR-FTIR) with a Bruker Alpha FTIR (Bruker, Karlsruhe, Germany) instrument, equipped with a ZnSe crystal. Spectra were recorded in the 600–4000 cm^−1^ range at room temperature (22 °C), with a spectral resolution of 4 cm^−1^ using a non-destructive measurement approach.

### 2.4. Cytogenetic Assay of Sta-MNPs on Triticum aestivum L. Root Tips

A preliminary assay was performed to assess the potential nanotoxicity effects of Sta-MNPs on environmental vegetation. Twenty intact wheat (*Triticum aestivum* L.) seeds were selected for each treated and control groups, based on uniformity in size and color and the absence of visible defects. Germination was carried out on moistened filter paper placed in sterile Petri dishes, under controlled laboratory conditions at Lucian Blaga University of Sibiu, at 22 ± 0.5 °C in complete darkness.

Four experimental groups were established: (i) the control group, supplied with 10 mL of distilled water; (ii) the starch control group, supplied with 10 mL of a 1% (*w*/*v*) aqueous starch solution; and (iii–iv) the Sta-MNP-treated groups, supplied with 10 mL of aqueous Sta-MNP suspensions at two dilution levels (100 and 200 µL/L, *v*/*v*), corresponding to nominal Fe_3_O_4_-equivalent concentrations of 0.559 and 1.118 mg/L, respectively. After 2–3 days, seeds exhibiting primary roots between 10 and 20 mm were selected for cytogenetic analysis, using all three individual root tips from each germinated seed.

Root tips were fixed for four hours in Carnoy’s fixative solution, consisting of glacial acetic acid and absolute ethanol in a 1:3 volume ratio, and subsequently preserved in 70% ethanol at 4 °C. For staining, the root apices were hydrolyzed successively in a 1N solution of HCl for 5 min and in a 1:1 solution of 37% HCl and distilled water for 20 min, followed by storage in modified carbol-fuchsin stain [27] for 24 h under refrigeration.

Microscope slides were prepared using the squash technique [28]. For each experimental condition, five slides were obtained from all three individual root tips of each germinated seed, which were mechanically dispersed on the slide with a drop of 45% acetic acid solution. The cytogenetic assessment involved the examination of between 1500 and 4500 number of cells per slide, distributed over more than 30 microscopic fields. All analyses were conducted by the same operator utilizing a Euromex IS 1153-EPL microscope (Euromex Optics, Arnhem, The Netherlands) with a 40× objective lens. Representative images of cellular abnormalities were obtained using a CMEX-18000-PRO digital camera and were processed with the Euromex Image Focus Alpha software (version x64).

The mitotic index (MI) and aberration index (AI) were evaluated as quantitative cytogenetic indicators according to Equations (5) and (6), respectively.

MI (%) = (TDC/TAC) × 100(5)

AI (%) = (TA/TAC) × 100(6)

where TAC represents the total number of analyzed cells, TA denotes the number of abnormal cells, and TDC corresponds to the total number of dividing cells.

### 2.5. Statistical Analysis

Data obtained from the microscopic examination of five slides corresponding to each experimental variant (control and Sta-MNP-treated samples) were expressed as mean ± standard deviation. Descriptive statistical analyses were performed for both the mitotic index (MI) and aberration index (AI) in all the experimental groups. Differences between the control and treated variants were evaluated using one-way analysis of variance (ANOVA), followed by Tukey’s Honest Significant Difference (HSD) post-hoc test for multiple pairwise comparisons. Statistical significance was set at *p* < 0.05. All statistical analyses and graphical representations were performed using Microsoft Excel (version 2016) and OriginLab software (version 2023b).

## 3. Results

### 3.1. The Results of Microstructural Property Investigations

**Figure 1:**
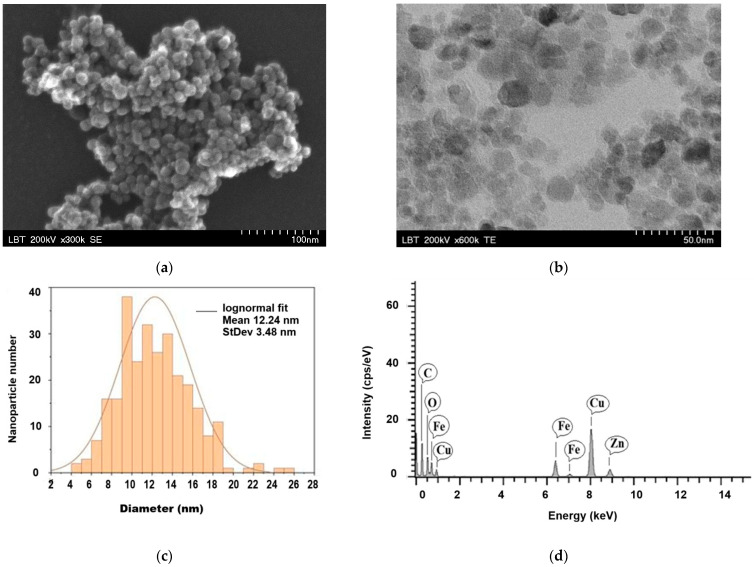
Morphological and elemental characterization of the synthesized starch-coated magnetite nanoparticles (Sta-MNP). (**a**) SEM image showing the morphology of the nanoparticles (scale bar: 100 nm); (**b**) TEM image (scale bar: 50 nm); (**c**) particle size distribution histogram obtained from TEM analysis with lognormal fitting; (**d**) EDS spectrum confirming the elemental composition of the synthesized nanoparticles.

The morphological and dimensional characterization of Sta-MNPs was carried out by transmission electron microscopy (TEM) and scanning electron microscopy (SEM). Images acquired through both techniques revealed a quasi-spherical geometry of the nanoparticles (Figure 1a,b), whereas the size distribution analysis was performed exclusively based on the TEM images. To this end, 373 nanoparticles were measured from five representative TEM images using the ImageJ software (version 1.8.0), and the resulting data were plotted as a histogram using the OriginLab software (version 2023b). Curve fitting using a lognormal function yielded a median value of 12.24 nm and a standard deviation of 3.48 nm, with diameters ranging from 4 to 26 nm (Figure 1c).

No significant particle agglomeration or anomalously large particles possibly suspected of ferrimagnetic characteristics were detected, which is consistent with the narrow hysteresis loop observed in the magnetization curve, as will be further discussed in the VSM section below. Nevertheless, the presence of isolated clusters cannot be entirely excluded, as will be subsequently evidenced by the NTA results presented in the following section, which are consistent with the multimodal distribution of the hydrodynamic diameter. The relatively small physical dimensions of the magnetic nanoparticles, combined with the absence of substantial agglomeration and the resulting colloidal stability in aqueous media, render the as synthetized Sta-MNP nanoparticles suitable candidates for biomedical applications.

The elemental composition of the Sta-MNPs was investigated using EDS, and the corresponding spectrum is presented in Figure 1d. The EDS spectrum confirmed the presence of Fe, O, and C, consistent with starch-coated iron oxide nanoparticles. The additional Cu and Zn peaks arose from the TEM support grid and associated background scattering during the EDS acquisition and were, therefore, not representative of the sample. Because the EDS analysis of polymer-coated nanoparticles does not provide reliable information on the stoichiometry of the iron oxide phase, phase identification was established using XRD, as discussed in the following section.

### 3.2. The Results of Crystalline Structure Investigations

**Figure 2:**
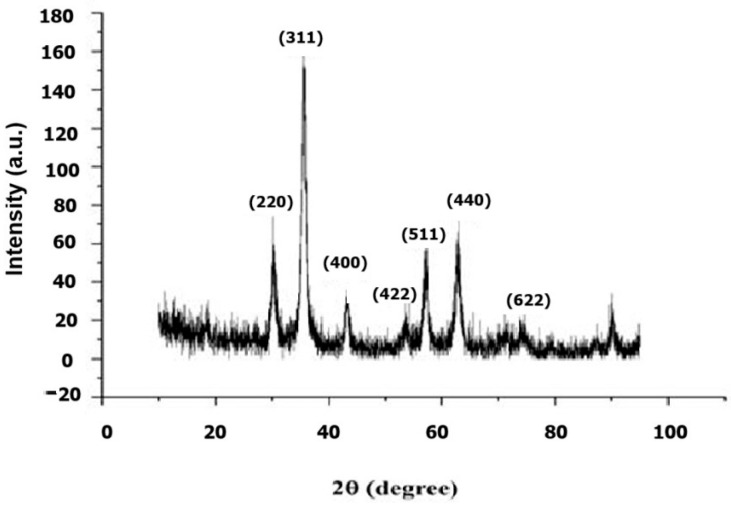
XRD pattern of the synthesized starch-coated magnetite nanoparticles (Sta-MNP), with the principal diffraction peaks indexed by their corresponding Miller indices (hkl). They indicate the formation of crystallites with typical diffraction peaks in accordance with JCPDS card no. 19-0629 that allow the calculation of the lattice parameter.

The crystalline structure of Sta-MNP was investigated using XRD, and the corresponding diffractogram is presented in Figure 2. The diffraction pattern revealed seven distinct Bragg reflection peaks, indexed to the (220), (311), (400), (422), (511), (440), and (622) crystallographic planes, all of which are characteristic of the inverse spinel structure of magnetite (Fe_3_O_4_), in accordance with JCPDS card no. 19-0629 [29]. The dominant reflection at the (311) plane further corroborated the formation of the Fe_3_O_4_ phase, with no additional peaks attributable to secondary phases or impurities detected. The interplanar spacing calculated for the (311) reflection was d_(311)_ = 0.252 nm, yielding a lattice parameter of a = 0.8363 nm, which is close to the value commonly reported for bulk magnetite (a = 0.8396 nm) [30]. However, considering the close structural similarity between magnetite and maghemite, X-ray diffraction alone does not allow their unequivocal discrimination. The mean crystallite size, determined from the most intense (311) reflection using the Scherrer equation, was estimated to be 8.60 nm. This value is smaller than the median physical diameter of 12.24 nm obtained from TEM analysis, a difference attributable to the presence of the starch coating layer surrounding the crystalline Fe_3_O_4_ core, which contributes to the overall physical dimensions of the nanoparticle as observed by TEM, while remaining undetected by XRD due to its amorphous nature.

### 3.3. The Results of Magnetic Property Investigations

**Figure 3:**
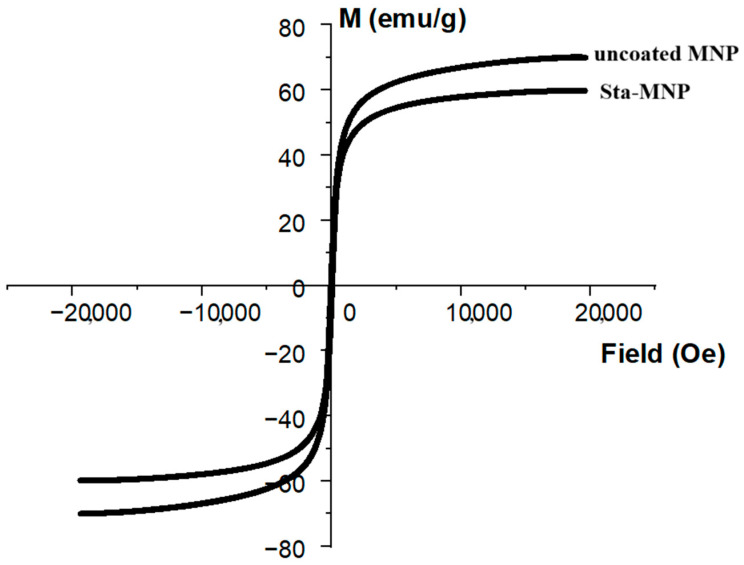
Room-temperature magnetization curves of the synthesized starch-coated magnetite nanoparticles (Sta-MNP) and uncoated magnetite nanoparticles (uncoated MNP). The magnetization curve of uncoated MNP tends to saturate at approx. 69 emu/g and presents a small coercivity, which is typical for superparamagnetic materials, while Sta-MNPs are also characterized by a high saturation magnetization of approx. 59 emu/g which is lower than that of uncoated MNNPs because of the organic shell.

The magnetic properties of Sta-MNPs were investigated by vibrating sample magnetometry (VSM), and the corresponding magnetization curve as a function of the applied magnetic field is presented in Figure 3. The saturation magnetization (Ms) was determined to be 59.81 emu/g, with a remanence (Mr) of approximately 0.69 emu/g and a coercivity (Hc) of 5.87 Oe, yielding a squareness ratio (Mr/Ms) of 0.012.

The magnetic core diameter, estimated from the Langevin function applied to the magnetization curve at room temperature and assuming spherical particle geometry, was determined to be 8.548 nm, in excellent agreement with the mean crystallite size of 8.60 nm obtained from XRD analysis, indicating that each nanoparticle behaves as a single magnetic domain coinciding with its crystalline core. The negligible values of remanence and coercivity, combined with the highly characteristic S-shaped hysteresis loop, are indicative of superparamagnetic behavior, consistent with the small crystallite size of 8.60 nm determined by XRD analysis, which is below the superparamagnetic size threshold typically reported for Fe_3_O_4_ nanoparticles (~25–30 nm) [31]. The uncoated MNPs exhibited a higher saturation magnetization (approximately 69.5 emu/g) than the Sta-MNPs. The lower saturation magnetization of the coated nanoparticles can be attributed to the presence of the non-magnetic starch shell, which diluted the magnetic content of the composite. The absence of significant hysteresis further confirms that Sta-MNPs exhibit no remanent magnetization upon removal of the external field, a property of considerable relevance for biomedical applications.

### 3.4. The Results of Colloidal Property Investigations

The colloidal stability and hydrodynamic behavior of the Sta-MNP aqueous suspension were evaluated through long-term sedimentation monitoring and nanoparticle tracking analysis (NTA). Periodic visual assessment of sedimentation, performed according to the methodology reported by Baki et al. (2021) [32] indicated that the suspension remained stable during storage at room temperature. After six months, only a minor sediment was observed at the bottom of the container, whereas vigorous mechanical agitation successfully restored suspension homogeneity, maintaining stability for an additional four months. However, by the eleventh month, redispersion was no longer achievable, and the system exhibited pronounced phase separation, indicating a loss of colloidal stability over prolonged storage.

**Figure 4:**
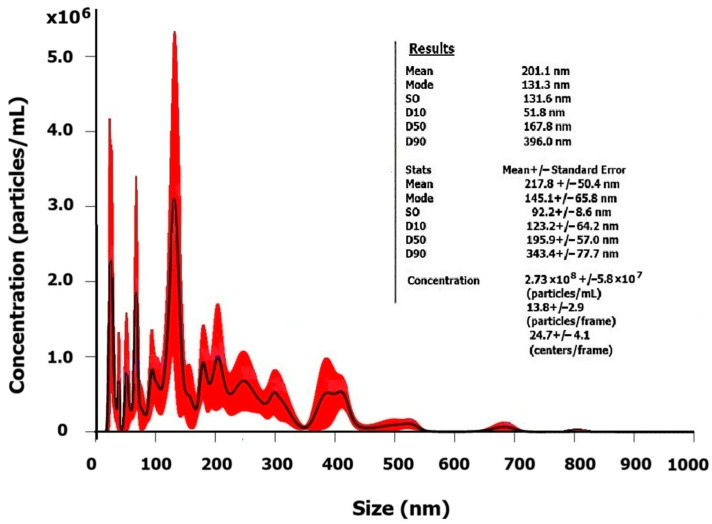
Hydrodynamic size distribution of Sta-MNPs determined by nanoparticle tracking analysis (NTA) at a dilution factor of 10^4^, expressed as concentration versus size (FTLA method). Inset: summary of statistical parameters obtained from NTA measurements. SO—standard output; D10—the cumulative distribution percentile denoting the particle size threshold at which 10% of the particles in the sample are smaller than the specified diameter; D50—the median particle size, D90—the cumulative distribution percentile corresponding to the particle size threshold at which 90% of the particles in the sample are smaller than the specified diameter.

The hydrodynamic size distribution determined by NTA is shown in Figure 4. Although VSM measurements confirmed the superparamagnetic nature of the individual nanoparticles, NTA revealed the existence of some hydrodynamic aggregates within the suspension, consistent with the multimodal distribution profile obtained. The analysis yielded a mean hydrodynamic diameter of 201.1 nm, a mode of 131.3 nm, and a standard deviation of 131.6 nm. The D10, D50, and D90 values were 51.8, 167.8, and 396.0 nm, respectively, demonstrating a broad particle size distribution encompassing multiple hydrodynamic sub-populations. The presence of a minor fraction of larger aggregates extending up to approximately 810 nm suggests the formation of hydrodynamic clusters, likely resulting from incomplete steric stabilization by the starch coating, which makes predictable the progressive loss of colloidal stability observed during long-term storage. Despite this aggregation tendency, the suspension maintained sufficient colloidal stability for 11 months before irreversible phase separation occurred. Taking into account the applied dilution factor of 10^4^, the particle concentration of the original suspension was estimated at 2.73 × 10^12^ particles/mL.

### 3.5. The Results of Surface Chemical Characterization

**Figure 5:**
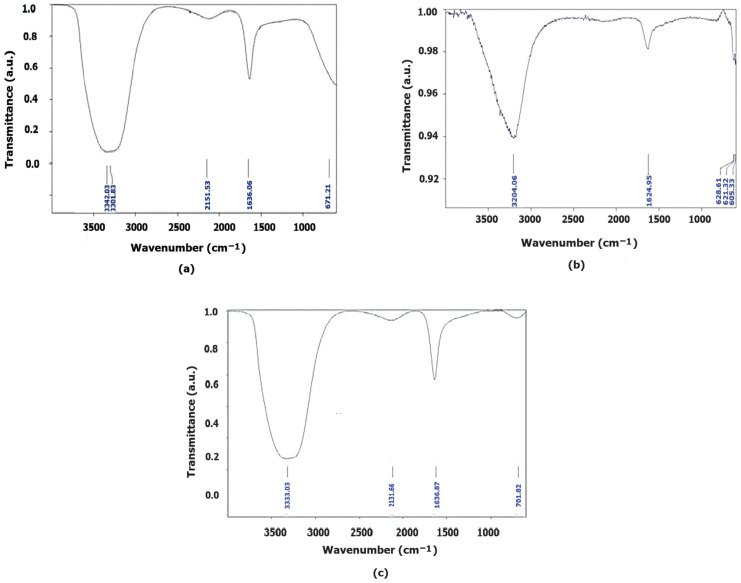
ATR-FTIR spectra of (**a**) Sta-MNP aqueous suspension recorded against air background; (**b**) Sta-MNP aqueous suspension recorded against water background; and (**c**) reference starch solution used in the synthesis (0.02 g/mL, recorded against air background).

The aqueous dispersion of starch-coated magnetite (Sta-MNP) was characterized by ATR-FTIR spectroscopy using two background configurations: air (Figure 5a) and water (Figure 5b). For comparison, the ATR-FTIR spectrum of the starch solution used in the synthesis (0.02 g/mL, air background) was also recorded (Figure 5c).

The ATR-FTIR spectrum of Sta-MNP recorded with air background (Figure 5a) showed a broad and intense absorption band in the region 3301–3342 cm^−1^, attributed to the stretching vibrations of the hydroxyl groups O–H in starch molecules, free water and adsorbed water, which appears shifted to 3204 cm^−1^ in the Sta-MNP spectrum recorded with water background (Figure 5b), and shifted to 3333 cm^−1^ in the spectrum of the reference starch solution (Figure 5c) [33,34], suggesting an extensive network of dynamic hydrogen bonds. These differences suggest mainly a modification of the hydrogen bonding network in the presence of nanoparticles, indicating an interaction between the hydroxyl groups of the starch coating and the Fe_3_O_4_ surface.

The band at 1636 cm^−1^, present in both air-background spectra, is attributed to the δ(H–O–H) bending vibration of bound water molecules associated with the hydrated starch coating surrounding the nanoparticles. A weak band at approximately 2131 cm^−1^, identified in both the Sta-MNP and starch solution spectra, was tentatively attributed to ubiquitous atmospheric CO_2_ that often is adsorbed to nanoparticles and polymers in suspension and was therefore not considered characteristic of the sample [35]. The band observed at 671 cm^−1^ is tentatively attributed to Fe–O stretching vibrations of the Fe_3_O_4_ core and the possible formation of Fe–O–C bonds at the nanoparticle surface. It should be noted that the characteristic Fe–O absorption bands of magnetite are typically reported in the 400–600 cm^−1^ region [36]; however, this range falls at or below the lower wavenumber limit of the instrument employed and could therefore not be fully resolved in the air background spectrum. In the fingerprint region (1000–1200 cm^−1^), a broad absorption consistent with the C–O–C stretching vibrations of the polysaccharide is observed, although no well-defined peaks are distinguished owing to band overlapping and the strong water absorption inherent to the aqueous colloidal system [33].

The spectrum recorded with water background (Figure 5b), in which the solvent contribution was subtracted, more clearly revealed the characteristic bands of magnetite. Three components were resolved in the 600–630 cm^−1^ region at 628, 621, and 605 cm^−1^, attributed to the Fe–O stretching vibrations of the spinel structure of Fe_3_O_4_, in agreement with values reported in the literature [36]. These bands were partially obscured in the air-background spectrum, where only a broader band at 671 cm^−1^ was apparent. The residual band at 1624 cm^−1^ reflects the incomplete subtraction of the water contribution, a phenomenon commonly encountered in ATR-FTIR measurements of aqueous systems [35]. The O–H band observed at 3204 cm^−1^ after water subtraction indicates that the water in the nanoparticle dispersion exhibits a hydrogen bonding structure different from that of pure water, with stretching frequencies shifted to lower wavenumbers, consistent with the structuring of water at the interface of the hydrophilic nanoparticles.

The absence of characteristic starch bands in the 900–1200 cm^−1^ region (C–O–C and C–O–H stretching and bending vibrations) across all three spectra can be explained by the low starch concentration in the dispersion and the strong water absorption in this spectral region, which obscures the polysaccharide signal [33]. Nevertheless, the presence of starch at the nanoparticle surface is indirectly supported by the modifications observed in the O–H band relative to the individual components.

In conclusion, ATR-FTIR analysis confirmed the presence of the Fe_3_O_4_ core through the characteristic Fe–O bands, while the comparison of the three spectra suggested an interaction between starch and the Fe_3_O_4_ nanoparticle surface, manifested through modifications of the hydrogen bonding network relative to the individual components.

### 3.6. The Results of Preliminary Nanotoxicity Investigations

**Figure 6:**
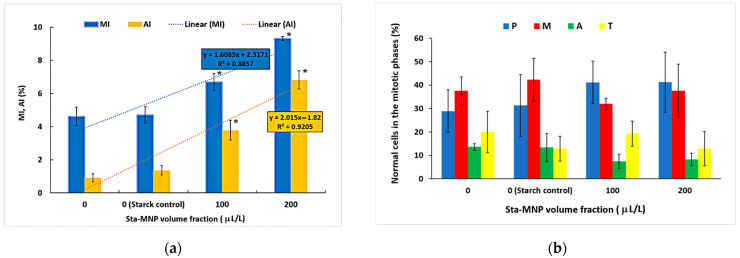
Cytogenetic effects of starch-coated magnetite nanoparticles (Sta-MNP) on *Triticum aestivum* L. root tip cells: (**a**) mitotic index (MI) and aberration index (AI) as a function of Sta-MNP volume fraction; (**b**) distribution of normal cells across the mitotic phases (prophase, P; metaphase, M; anaphase, A; telophase, T) in controls and Sta-MNP-treated samples. Data are presented as mean ± standard deviation (SD) (*n* = 5). Statistical significance relative to the control is indicated by * (*p* < 0.01), according to a one-way ANOVA followed by Tukey’s HSD test.

The mitotic index (MI) and aberration index (AI) determined for *Triticum aestivum* L. root tip cells exposed to Sta-MNP at two volume fractions are presented in Figure 6a. The results demonstrated a concentration-dependent increase in both cytogenetic indicators. The MI increased significantly with increasing Sta-MNP concentration, reaching its highest value at 200 µL/L, and exhibited a positive linear relationship with the Sta-MNP volume fraction (R^2^ = 0.8857). A similar concentration-dependent trend was observed for the AI, which also showed a linear correlation with the Sta-MNP volume fraction (R^2^ = 0.9205) (Figure 6a). Both the mitotic and aberration indices increased with increasing Sta-MNP concentrations, indicating that enhanced cell proliferation was accompanied by a higher frequency of chromosomal abnormalities. These results suggest that stimulation of mitotic activity occurred together with measurable cytogenetic alterations under the experimental conditions used.

The starch control group demonstrated cytogenetic parameters similar to those of the control group, with a mitotic index (MI) of 4.71 ± 0.49% and an aberration index (AI) of 1.36 ± 0.27%, showing no statistically significant differences between the control groups. Additionally, the distribution of cells across the various mitotic phases was consistent with that of the control group, with no statistically significant differences observed in the proportions of cells in the prophase, metaphase, anaphase, or telophase. These results suggest that the starch solution alone did not induce detectable cytogenetic alterations or disrupt normal mitotic progression under the experimental conditions. Figure 6b illustrates the distribution of normal cells across the various mitotic phases. Exposure to Sta-MNP altered the distribution of normal cells among the mitotic phases (Figure 6b). Compared with the control, the treated root meristem cells exhibited a higher proportion of cells in prophase and reduced proportions of cells in anaphase and telophase, suggesting altered progression through mitosis.

Overall, the cytogenetic data suggest that Sta-MNP exposure has a dual impact on mitotic activity in *Triticum aestivum* L. root tip cells. The volume fraction-dependent increase in the mitotic index (MI) implies stimulation of cell cycle entry, while the accumulation of cells in prophase and the reduction in anaphase and telophase fractions indicate a disruption in normal mitotic progression, likely due to an impediment at the prophase-to-metaphase transition. This mitotic disruption is further supported by the more pronounced increase in aberration index (AI), compared to MI, and suggests that the induction of chromosomal aberrations goes beyond general proliferative stimulation. Nonetheless, the maximum AI value recorded at the highest volume fraction appears to correspond to mild to moderate cytogenotoxic effects within the tested volume fraction range (0–200 µL/L). Collectively, these findings show that Sta-MNP exposure simultaneously promotes cell division and mildly compromises mitotic fidelity, a pattern consistent with proliferative activation associated with moderate cytogenotoxic disturbance. Further investigation into the underlying mechanisms and dose-dependency at higher concentrations and longer exposure periods is warranted.

The types and relative frequencies of chromosomal aberrations recorded in *Triticum aestivum* L. root-tip cells are presented in Table 1.

**Figure 7:**
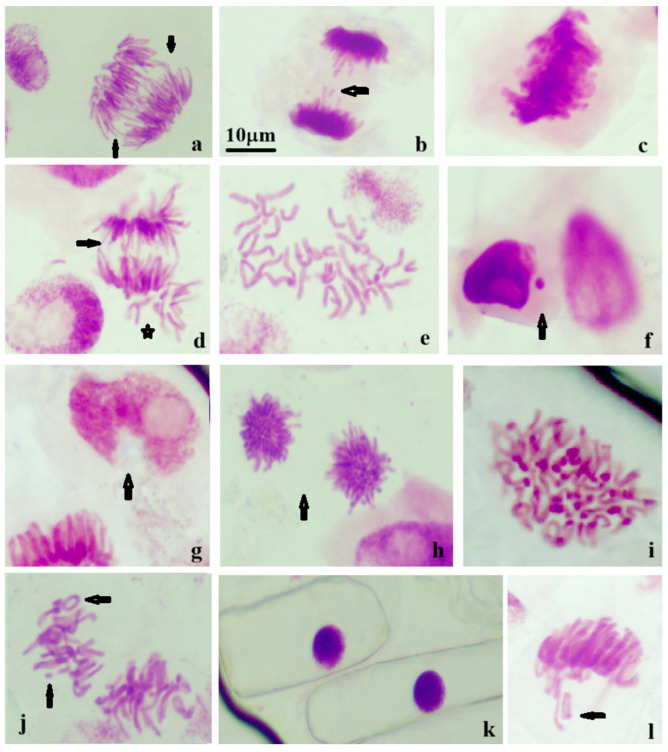
Representative photomicrographs of chromosomal aberrations observed during cytogenetic analysis: (**a**) multiple bridges in anaphase (200 µL/L); (**b**) chromosomal fragment in anaphase (100 µL/L); (**c**) sticky metaphase (100 µL/L); (**d**) anaphase with bridges (arrow) and expelled chromosomes (star) (100 µL/L); (**e**) C-mitosis (100 µL/L); (**f**) micronucleus (100 µL/L); (**g**) nuclear lesion (100 µL/L); (**h**) star anaphase (200 µL/L); (**i**) disrupted metaphase (100 µL/L); (**j**) asymmetric anaphase with chromosomal fragment and ring chromosome (100 µL/L); (**k**) hyperchromasia (200 µL/L); (**l**) metaphase with vagrant chromosome (200 µL/L). Scale bar: 10 µm. Arrows and stars indicate the specific chromosomal aberrations.

In the control samples, the spontaneous aberration spectrum was dominated by bridges, vagrant chromosomes and disrupted metaphases, whereas nuclear lesions and hyperchromasia were virtually absent (Table 1). Following Sta-MNP exposure, the overall frequency of chromosomal aberrations increased, as reflected by the progressive increase in the aberration index. However, the different types of chromosomal aberrations exhibited distinct response patterns. In contrast to chromosome segregation abnormalities, the most pronounced response was observed for nuclear aberrations, particularly nuclear lesions and hyperchromasia, both of which increased markedly compared to the control. Vagrant chromosomes showed a clear concentration-dependent increase, whereas disrupted metaphases became more frequent, especially at the highest Sta-MNP concentration (Table 1). The principal types of chromosomal aberrations identified during cytogenetic screening are illustrated by representative photomicrographs in Figure 7.

## 4. Discussion

The physicochemical properties of Sta-MNPs are broadly consistent with those reported in the literature for starch-coated iron oxide nanoparticles synthesized by co-precipitation, although notable differences exist depending on the specific synthesis conditions employed. The median physical diameter of 12.24 nm determined by TEM in the present study is smaller than the values reported by Dung et al. (2009) [37], who obtained an average TEM diameter of approximately 20 nm for starch-coated magnetite nanoparticles, the 21.61 nm reported by Elkhenany et al. (2020) [38] for starch-coated Fe_3_O_4_ nanoparticles, and the value of approximately 21 nm reported by Zheng et al. (2018) for starch-stabilized Fe_3_O_4_ nanoparticles [39]. The smaller physical dimensions of Sta-MNP may be attributed to differences in synthesis parameters, particularly the Fe^2+^/Fe^3+^ ratio, reaction temperature, pH, and starch concentration, all of which influence nucleation and growth kinetics during co-precipitation. The quasi-spherical morphology observed in this study is consistent with that reported by Dung et al. (2009) [37] and Elkhenany et al. (2020) [38]. Krasitskaya et al. (2022) reported cubic nanocrystals with an average size of approximately 11.5 nm [12]. Robinson et al. (2019) [40] demonstrated that the particle morphology and crystallite size of starch-functionalized magnetite nanoparticles are strongly dependent on the synthesis route employed, with crystallite sizes ranging from approximately 14 nm for co-precipitation to 67 nm for oxidation–precipitation in the absence of starch, highlighting the critical role of synthesis conditions in controlling particle dimensions. Furthermore, Hajalilou et al. (2024) [41] demonstrated that the choice of reducing agent during co-precipitation significantly influences both particle morphology and size, with NaOH producing larger, more cubic-shaped nanoparticles than the quasi-spherical nanoparticles obtained with NH_4_OH, with average crystallite sizes ranging from approximately 7 to 14 nm depending on the coating and reducing agent employed.

The mean crystallite size of 8.60 nm determined by XRD and the magnetic core diameter of 8.548 nm obtained from Langevin function fitting are in excellent agreement, confirming the single-domain nature of the nanoparticles with an average coating shell of 3.6 nm. Dung et al. (2009) [37] estimated the magnetic core diameter to be approximately 14 nm, with a starch layer thickness of approximately 3 nm, noting that the magnetic core diameter was close to the crystallite size determined by XRD, which is consistent with our observations. The smaller magnetic core diameter obtained in the present study relative to that reported by Dung et al. (2009) is consistent with the smaller overall physical dimensions of the Sta-MNP [37].

The saturation magnetization of 59.81 emu/g obtained for Sta-MNP is higher than the range of 30–50 emu/g reported by Dung et al. (2009) [37] for starch-coated magnetite nanoparticles, and substantially higher than the value of 1.14 emu/g reported by Elkhenany et al. (2020) [38] for starch-coated Fe_3_O_4_ nanoparticles. The markedly lower Ms reported by Elkhenany et al. (2020) may be attributed to the significant reduction in magnetization induced by starch coating, as the authors noted that the starch layer hinders magnetization in the presence of an external magnetic field [38]. Hajalilou et al. (2024) reported saturation magnetization values of 53–67 emu/g for starch-coated Fe_3_O_4_ nanoparticles of similar mean dimensions (11.2 nm) synthesized by co-precipitation [41], which are in good agreement with those obtained in the present study. The authors also confirmed superparamagnetic behavior for all starch-coated samples and noted that the reduction in Ms relative to that of the bare nanoparticles was attributed to the non-magnetic contribution of the starch coating layer to the overall particle mass, a finding consistent with the study of Piosik et al. (2021) [42], who reported that the saturation magnetization of native starch-coated Fe_3_O_4_ nanoparticles was reduced by 46 emu/g compared to that of the uncoated counterparts. In contrast, Krasitskaya et al. (2022) [12] reported a saturation magnetization of 29.8 emu/g for starch-coated iron oxide nanoparticles of cubic morphology and similar size (11.5 nm), a value approximately half of that obtained in the present study, likely reflecting the influence of particle morphology and crystalline phase on the magnetic response. It is worth noting that the effect of starch coating on saturation magnetization is not straightforward and depends strongly on the synthesis conditions and particle size distribution, as demonstrated by Šuljagić et al. (2021) [43] and Lazarević et al. (2025) [44] for starch-coated CoFe_2_O_4_ nanoparticles, where both increases and decreases in Ms were observed depending on the synthesis method employed. The negligible remanence and coercivity values confirmed superparamagnetic behavior, consistent with the findings of Dung et al. (2009) [37], Krasitskaya et al. (2022) [12], and Hajalilou et al. (2024) [41] for starch-coated nanoparticles of comparable dimensions.

ATR-FTIR analysis confirmed the presence of the starch coating on the nanoparticle surface, in agreement with the findings reported by Dung et al. (2009) [37], who demonstrated that starch is chemisorbed onto the magnetite nanoparticle surface through hydroxyl group interactions with Fe atoms. Elkhenany et al. (2020) [38] similarly confirmed starch coating through FTIR analysis, identifying characteristic peaks at 1028–1033 cm^−1^, corresponding to the interaction between starch and Fe atoms. The O-H stretching vibrations observed in the Sta-MNPs at 3342 and 3301 cm^−1^ compared to 3333 cm^−1^ in the reference spectrum of starch indicate that the O–H group acts as a hydrogen bond donor to the metal surface shifting the broad band which is concordant with the reports of Dung et al. (2009) [37] and Krasitskaya et al. (2022) [12]. The presence of the starch coating was further corroborated by characteristic absorption bands in the fingerprint region, consistent with the FTIR findings reported for starch-functionalized magnetic nanoparticles by Šuljagić et al. (2021) [43] and Lazarević et al. (2025) [44], who similarly confirmed starch attachment through hydroxyl group interactions with the metal oxide surface.

The multimodal hydrodynamic size distribution observed by NTA, with a mean diameter of 201.1 nm, is consistent with the tendency of starch-coated Fe_3_O_4_ nanoparticles to form hydrodynamic aggregates in aqueous suspension, as previously reported. Zheng et al. (2018) [39] reported a mean hydrodynamic diameter of 217.9 ± 8.2 nm for starch-stabilized Fe_3_O_4_ nanoparticles determined by dynamic light scattering (DLS), a value comparable to that obtained in the present study, despite the differences in synthesis conditions and measurement technique. The significantly larger hydrodynamic diameter relative to the physical diameter determined by TEM is explained by the well-established behavior of starch-coated nanoparticles in aqueous media, where steric stabilization by the hydrated polysaccharide shell and the formation of loosely bound hydrodynamic clusters contribute to the apparent increase in the particle size. Elkhenany et al. (2020) [38] similarly noted that the DLS-measured hydrodynamic diameters were substantially larger than the TEM-derived physical diameters, attributing this discrepancy to the contribution of the dispersing solution to the hydrodynamic diameter measurement. The colloidal stability of the Sta-MNP suspension was maintained for up to 11 months before irreversible phase separation occurred, which compares favorably with the six-month stability reported by Dung et al. (2009) [37] for starch-coated magnetite nanoparticles stored under ambient conditions, suggesting that the synthesis conditions employed in the present study may have yielded a more stable polysaccharide coating.

The hydrodynamic diameter determined by NTA was larger than the particle size observed by TEM, as usually expected, as it is a measure of the particle in motion inside a liquid, capturing the core plus any attached surface ligands, surfactants (such as starch), and the tightly bound layer of solvent molecules. The presence of low-intensity peaks in the concentration versus hydrodynamic diameter graph reflects a certain polydispersity, also highlighted at the level of the physical diameter obtained by TEM, and may also indicate the presence of small nanoparticle aggregates in the aqueous suspension. Such aggregation is commonly observed in colloidal systems and may influence nanoparticle transport, sedimentation behavior, and interactions with plant tissues, thereby affecting their bioavailability. Nevertheless, the hydrodynamic size distribution remained within the nanoscale range, suggesting that the starch coating provided sufficient colloidal stabilization to maintain a suspension suitable for the biological experiments performed in this study. The cytogenetic effects of Sta-MNP observed in the present study are broadly consistent with those reported in the literature for iron oxide nanoparticles in plant model systems, although notable differences exist depending on the nanoparticle composition, surface coating, concentration, and plant species investigated. Iron oxide nanoparticles have been reported to exert concentration-dependent effects on plant biology, with beneficial effects at low concentrations and toxic effects at high concentrations, as reviewed by Bhatia et al. (2025) [45]. Iron oxide nanoparticles may exert genotoxic effects through primary and secondary mechanisms, as described by Madhyastha et al. (2024) [46]. Primary genotoxicity may involve the direct interaction of nanoparticles with genetic material and proteins, whereas indirect primary genotoxicity has been associated with the generation of reactive oxygen species (ROS), which may induce oxidative damage to DNA and proteins [46]. Previous studies have suggested that these processes can interfere with cell cycle regulatory pathways, mitotic spindle organization, and DNA repair mechanisms, ultimately contributing to chromosomal abnormalities [46]. The treatment-dependent increase in MI observed in *Triticum aestivum* L. root tip cells is in contrast with the results reported by Tasar (2023) [17] and Kizilkaya et al. (2023) [47], who found significant decreases in MI in plant root meristematic cells exposed to uncoated Fe_2_O_3_ nanoparticles, with MI values as low as 6.06% at 250 µg/mL in *Allium cepa* root tip cells, accompanied by pronounced increases in chromosomal aberration frequency, reaching up to 62.03%. The maximum AI value of 6.82 ± 0.55% recorded for Sta-MNP at 200 µL/L was substantially lower than those reported for uncoated Fe_2_O_3_ nanoparticles by Kizilkaya et al. (2023) [47] and Tasar (2023) [17]. However, this comparison should be interpreted with caution because the studies differ in terms of nanoparticle composition, surface chemistry, concentration range, and plant model. Nevertheless, the lower cytogenetic response observed for Sta-MNPs is consistent with previous reports, indicating that polysaccharide coatings may improve colloidal stability and reduce nanoparticle interactions with biological systems. This difference may also reflect variations in the nanoparticle composition, surface chemistry, particle size, exposure concentration, and biological model employed. Importantly, the absence of significant differences between the starch control and distilled water control indicates that the starch stabilizing matrix alone did not measurably affect the investigated cytogenetic endpoints. Therefore, the cytogenetic alterations observed following Sta-MNP exposure are more likely associated with nanoparticle-containing suspensions than with the starch coating itself. Zheng et al. (2018) [39] demonstrated that starch coating significantly reduced the genotoxic effects of Fe_3_O_4_ nanoparticles compared to their uncoated counterparts in zebrafish gill and liver tissues, attributing this mitigation to the steric stabilization provided by the polysaccharide layer, which is consistent with the comparatively moderate cytogenetic effects observed for the starch-coated nanoparticles in the present study.

The increase in the mitotic index observed in the present study, together with the altered distribution of mitotic phases, indicates that Sta-MNP exposure affects mitotic progression. Similar changes in mitotic parameters have been reported for other surface-coated iron oxide nanoparticle formulations in plant systems. The mild to moderate cytogenotoxic effects observed in the present study are consistent with our other results reported for TMA-stabilized iron oxide nanoparticles of comparable dimensions on *Zea mays* root tip cells, where a maximum AI of 1.81% was observed, a lower value relative to the present study, likely reflecting differences in nanoparticle surface chemistry, plant species sensitivity and exposure conditions [48]. The types of chromosomal changes recorded in the present study, including C-mitosis, star anaphase, sticky chromosomes, vagrant chromosomes, bridges, and nuclear lesions, were consistent with the aberration spectra reported for iron oxide nanoparticles in various plant bioassay systems. C-mitosis and other spindle-related aberrations are associated with disturbances in the mitotic apparatus, whereas chromosomal bridges, fragments, and nuclear lesions are linked to oxidative DNA damage in previous studies [46]. Although these mechanisms may contribute to the observed abnormalities, they were not directly examined in the present study. As noted by Kizilkaya et al. (2023) [47], even nanoparticles with the same chemical composition may behave differently in biological systems depending on their primary size, size distribution, hydrodynamic diameter, and surface chemistry, underscoring the importance of comprehensive physicochemical characterization when interpreting cytogenetic data. Taken together, the increase in the mitotic index, redistribution of mitotic phases, and moderate increase in chromosomal aberrations indicate that Sta-MNP exposure affects normal mitotic progression while inducing measurable cytogenetic alterations. Further studies employing complementary molecular approaches are required to clarify the mechanisms underlying these observations and evaluate the effects of higher nanoparticle concentrations and longer exposure periods.

The comprehensive physicochemical characterization of Sta-MNPs, combined with the cytogenetic assessment performed in the present study, provides complementary information relevant to their biological evaluation and supports further studies aimed at assessing their suitability for use in biomedical and agricultural applications. The superparamagnetic behavior, high saturation magnetization, and colloidal stability of the Sta-MNPs are characteristics that may be advantageous for such applications. Although the moderate cytogenetic alterations observed at the tested concentrations suggest a favorable biological response under the present experimental conditions, additional studies involving broader concentration ranges, different biological models, and greenhouse or field conditions are required before their safety and practical applicability can be established.

The cytogenetic responses observed in this study further demonstrate the usefulness of the *Triticum aestivum* assay as a sensitive screening tool for detecting early biological effects induced by starch-coated magnetite nanoparticles. Alterations in mitotic activity and chromosomal integrity contribute to the preliminary identification of genotoxic hazards and complement other approaches used in nanomaterial safety assessments. However, additional investigations using mammalian test systems are required before conclusions can be drawn regarding potential human health risks.

## 5. Conclusions

In this study, starch-coated magnetite nanoparticles (Sta-MNPs) were successfully synthesized via co-precipitation and comprehensively characterized using a complementary set of analytical techniques. The results confirmed the successful formation of stable, superparamagnetic magnetite nanoparticles with a starch coating effectively bound to the particle surface and good colloidal stability during storage at room temperature. Preliminary cytogenetic assessment of *Triticum aestivum* L. root tip cells demonstrated treatment-dependent changes in mitotic activity and an increase in chromosomal aberrations across the investigated concentration range (0–200 µL/L). This indicates that Sta-MNPs influence cell division under the experimental conditions applied. These findings warrant further investigation over a broader concentration range and for longer exposure periods. The integration of comprehensive physicochemical characterization with cytogenetic assessment represents an important strength of the present study, providing complementary information for understanding the relationship between the physicochemical properties of nanoparticles and their biological responses in plant systems. Collectively, the results of this study indicate that starch-coated magnetite nanoparticles combine favorable physicochemical and magnetic properties with a relatively favorable cytogenetic response under the experimental conditions investigated. These findings support the further evaluation of starch-coated magnetite nanoparticles for biomedical and agricultural applications, highlighting the need for additional studies to comprehensively assess their biological safety and practical applicability.

## Figures and Tables

**Table 1:** Relative frequencies (mean ± SD, %) of chromosomal aberration types observed in *Triticum aestivum* L. root tip cells following exposure to different volume fractions of starch-coated magnetite nanoparticles (Sta-MNP). The starch control represents the sample treated with only a 1% starch solution.

Aberration Type		Control (%)	Starch Control (%)	100 µL/L (%)	200 µL/L (%)
	Nuclear lesions	0.000 ± 0.000	0.000 ± 0.000	0.928 ± 0.536	1.311 ± 0.288
Nuclear aberrations	Hyperchromasia	0.016 ± 0.005	0.254 ± 0.165	1.003 ± 0.504	2.260 ± 1.157
	Micronuclei	0.045 ± 0.064	0.052 ± 0.040	0.069 ± 0.042	0.181 ± 0.076
	Binucleate cells	0.057 ± 0.040	0.056 ± 0.041	0.207 ± 0.113	0.196 ± 0.076
	Breaks/fragments	0.061 ± 0.016	0.073 ± 0.042	0.166 ± 0.130	0.174 ± 0.055
Structural aberrations	Bridges	0.189 ± 0.124	0.146 ± 0.062	0.185 ± 0.156	0.275 ± 0.050
	Ring chromosomes	0.000 ± 0.000	0.000 ± 0.000	0.004 ± 0.001	0.006 ± 0.003
	Gaps	0.000 ± 0.000	0.015 ± 0.009	0.034 ± 0.007	0.134 ± 0.064
	Asymmetry	0.000 ± 0.000	0.017 ± 0.015	0.028 ± 0.004	0.128 ± 0.073
	Vagrant chromosomes	0.152 ± 0.037	0.231 ± 0.143	0.337 ± 0.167	0.646 ± 0.080
Segregation aberrations	Laggard chromosomes	0.071 ± 0.008	0.075 ± 0.045	0.047 ± 0.041	0.262 ± 0.084
	Delayed anaphase	0.097 ± 0.050	0.216 ± 0.184	0.121 ± 0.058	0.153 ± 0.065
	C-mitosis	0.047 ± 0.015	0.050 ± 0.031	0.265 ± 0.147	0.174 ± 0.148
	Stickiness	0.010 ± 0.013	0.192 ± 0.121	0.136 ± 0.099	0.175 ± 0.045
Condensation aberrations	Star anaphase	0.010 ± 0.013	0.017 ± 0.012	0.028 ± 0.018	0.124 ± 0.047
	Disrupted metaphase	0.153 ± 0.082	0.074 ± 0.069	0.222 ± 0.128	0.618 ± 0.374

## Data Availability

All data are contained within the article.
